# Vascular Complication after Collagenase Injection and Manipulation for Dupuytren’s Contracture: A Case Report

**DOI:** 10.31662/jmaj.2018-0063

**Published:** 2019-05-16

**Authors:** Yusuke Kawano, Takeshi Hagiwara, Nobuyuki Tanaka, Takumi Nakamura, Hiroki Takeda, Tadashi Itabashi, Mitsuru Furukawa, Kentaro Kikuchi, Kunimasa Okuyama

**Affiliations:** 1 Department of Orthopaedic Surgery, Shizuoka City Shimizu Hospital, Shizuoka, Japan

**Keywords:** Dupuytren’s contracture, collagenase, vascular complication, Raynaud’s phenomenon

## Abstract

A case of a vascular complication after collagenase injection and manipulation for Dupuytren’s contracture in a 57-year-old Japanese man is described. The patient presented with a 10-year history of worsening primary Dupuytren’s contracture. The metacarpophalangeal joint of his left little finger had a flexion contracture of 40° and was treated by collagenase injection. When the patient returned to our hospital for manipulation 24 hours later, however, his left little finger was almost completely improved because he hit his finger on the car’s gear lever. Then, 9 months after collagenase injection, in the first winter, he complained of a painful and pale left little finger occurring a few times a day, lasting for about 10 minutes. Now, two years after collagenase injection, the episodes of Raynaud’s phenomenon remain. Although Raynaud’s phenomenon after collagenase injection and manipulation for Dupuytren’s contracture is considered rare, it is a complication to be noted.

## Introduction

Collagenase clostridium histiolyticum (CCH) has been established as one of the options for treating Dupuytren’s contracture in Europe and the United States ^(1), (2)^, and it has been available in Japan since September 2015. Although there are many reports about collagenase injection, few reports of its complications exist ^(3), (4)^. A case of what seemed to be a vascular complication following collagenase injection for Dupuytren’s contracture is reported.

## Case Report

**Figure 1.**
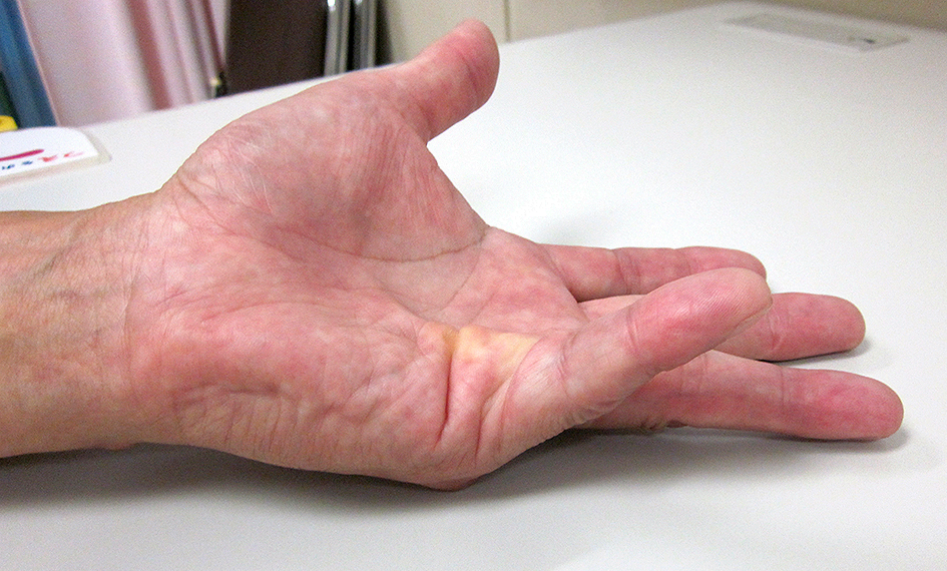
The metacarpophalangeal joint of the patient’s left little finger has a 40° flexion contracture.

A 57-year-old man had a flexion contracture in the metacarpophalangeal (MP) joint of his left little finger for 10 years. Although he quit smoking cigarettes 10 years earlier, he smoked 40 cigarettes a day for 20 years. There was no particular past medical history. He presented to our hospital with a palpable cord at the proximal MP joint of his little finger that caused a 40° contracture at the only MP joint (Figure 1). After receiving an explanation of the injection and surgical therapies, the patient requested injection therapy. Collagenase (0.58 mg, XIAFLEX, Asahikasei Pharma, Tokyo, Japan) was injected proximal to the metacarpophalangeal joint crease into the palpable cord.

**Figure 2.**
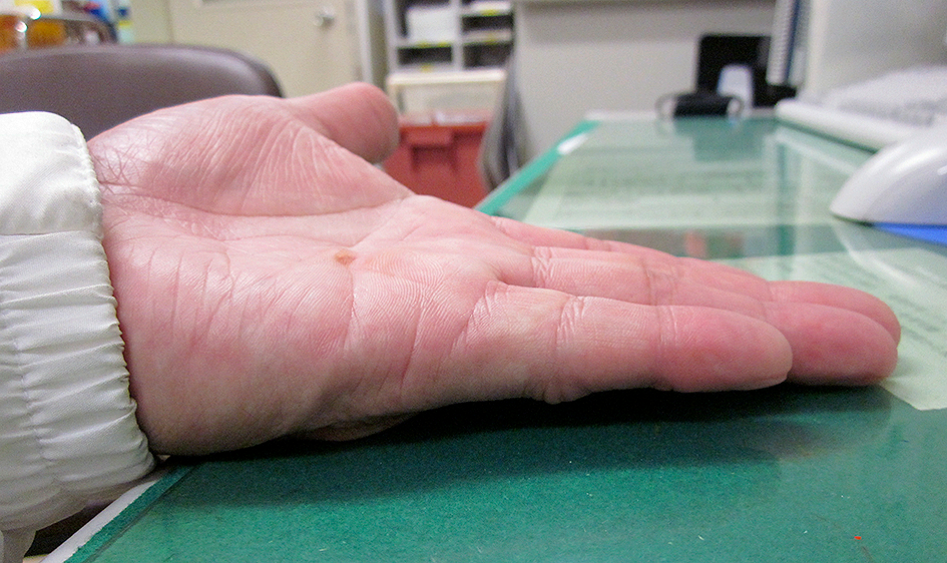
Three months after CCH injection, the affected joint is almost fully extended.

**Figure 3.**
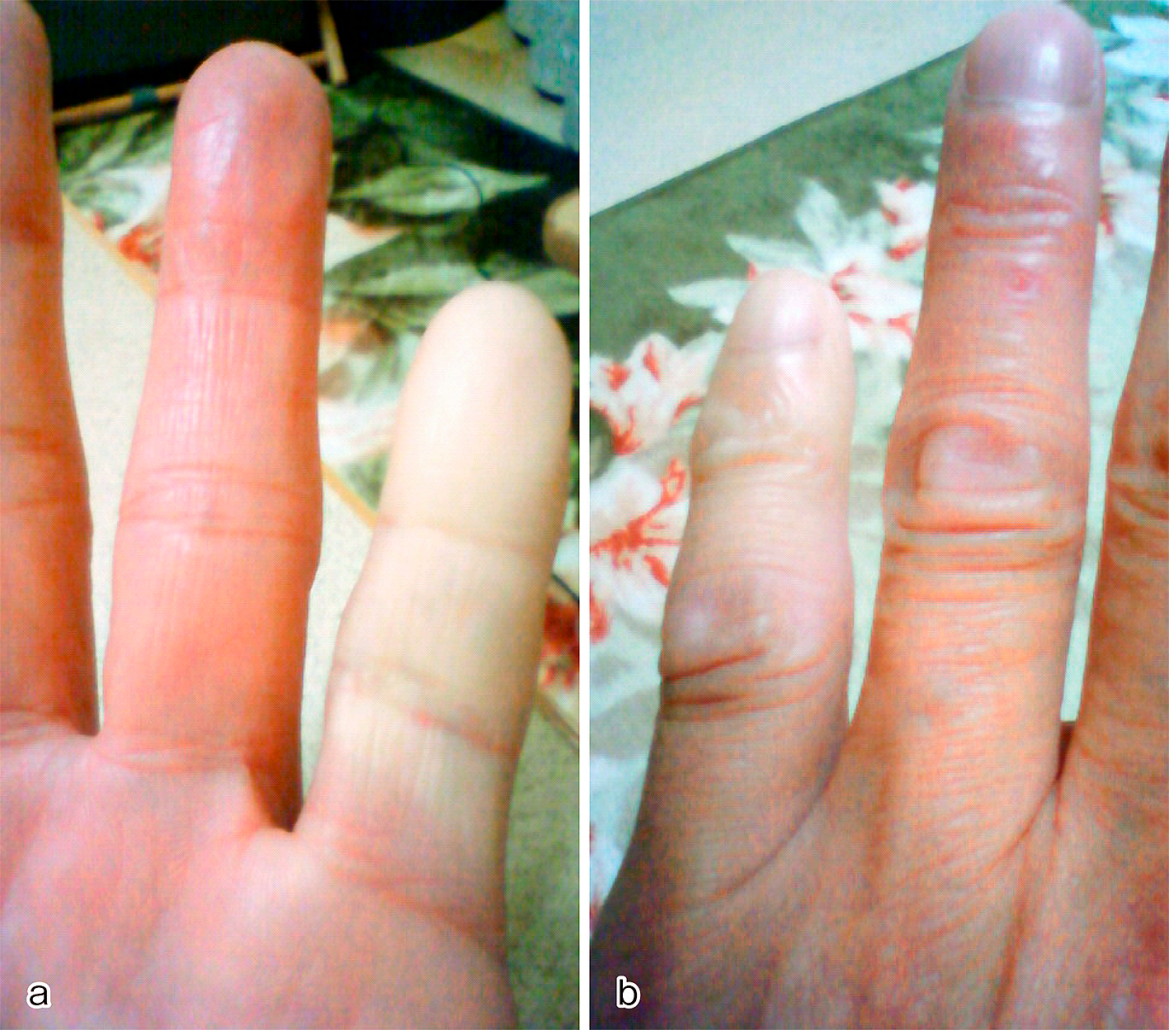
This picture was provided by the patient. In this picture, his left little finger is pale: a) shows the volar side, and b) is the dorsal side.

Manipulation was scheduled to take place 24 hours after the injection. When he came back to our hospital for manipulation 24 hours later, however, his left little finger was almost completely improved because he hit his finger on his car’s gear lever. He had little finger pain without skin laceration. Three months after the injection, he could fully extend the MP joint of his left little finger (Figure 2). However, nine months after collagenase injection, in the first winter, he complained of a painful and pale left little finger occurring a few times a day, lasting for about 10 minutes. He mentioned that he had never experienced such symptoms before, and he showed a picture (Figure 3) that was taken when his left little finger was painful and pale. Based on the history and the picture he showed, Raynaud’s phenomenon was diagnosed. Currently, two years have passed, and he complained that the symptoms became progressively worse when it was cold. The patient agreed that all data will be published.

## Discussion

A double-blind, randomized, and controlled study of CCH in the USA, Europe, and Australia has already been performed (CORD-I and CORD-II) ^(5), (6)^. According to CORD-I and CORD-II ^(5), (6)^, no patients experienced Raynaud’s phenomenon. Although 98% of the patients experienced treatment-related adverse events in reports of the Japanese CCH clinical trials (CORD-J) ^(7)^, no patients experienced Raynaud’s phenomenon. When reviewing CCH for introduction to Japan, the Pharmaceuticals and Medical Devices Agency in Japan used CORD-II as the reference ^(6)^. There was no description of Raynaud’s phenomenon in the article on CORD-II mentioned earlier ^(6)^, but internal documents showing the results of a more detailed analysis of CORD-II noted it in 2 of 63 cases ^(8)^, and a causal relationship was ruled out. In clinical studies, Spiers et al reported one case of Raynaud’s phenomenon after CCH injection ^(4)^. They suggested that follow-up studies following collagenase treatment should report any vascular complications.

In the present case, although there may have been a disturbance of peripheral blood vessels due to the rapid extension of the finger when it hit the car’s gear lever, in addition to the fact that the patient was a heavy smoker having an effect, it is thought that a causal relationship with CCH cannot be ruled out. Since administration of CCH caused inflammation at the administration site and capillary failure, cold-induced vasodilation did not occur, and Raynaud’s phenomenon may have occurred. Cold-induced vasodilation (CIVD) generally occurs 5–10 min after the start of local cold exposure of the extremities. This phenomenon is believed to reduce the risk of local cold injuries. CIVD is probably caused by a sudden decrease in the release of neurotransmitters from the sympathetic nerves to the muscular coat of the arteriovenous anastomoses (AVAs) due to local cold. AVAs are specific thermoregulatory organs that regulate blood flow in the cold and heat ^(9)^. In a study using CCH, the pathological findings showed destruction of the perineurium and the small vein ^(10)^. Thus, we believe that the administration of CCH was involved in the Raynaud’s phenomenon, in the present case. However, the CCH administration cannot explain the gradual deterioration of Raynaud’s phenomenon, but the blood vessel may have been vulnerable due to the patient’s past history of heavy smoking.

To the best of our knowledge, there are few reports of vascular complications after CCH injection for Dupuytren’s contracture in an Asian patient. We believe that it is necessary to report all cases of further similar adverse events.

## Article Information

### Conflicts of Interest

None
